# Robotic diaphragm plication: functional and surgical outcomes of a single-center experience

**DOI:** 10.1007/s00464-023-09942-7

**Published:** 2023-03-13

**Authors:** Uyen-Thao Le, Laurin Titze, Petar Hundeshagen, Bernward Passlick, Severin Schmid

**Affiliations:** Department of Thoracic Surgery, Medical Center, University of Freiburg, Hugstetter Straße 55, 79106 Freiburg, Germany

**Keywords:** Diaphragm elevation, Diaphragm plication, Robot-assisted thoracoscopic surgery

## Abstract

**Background:**

Diaphragm plication remains the only effective treatment for diaphragm paralysis. Robot-assisted thoracoscopic (RATS) diaphragm plication combines advantages of open and thoracoscopic techniques. We present our experiences focussing on lung-function improvement and surgical outcome.

**Methods:**

In this single-center retrospective study with comparative analysis, perioperative data of all patients who underwent RATS or thoracoscopic (VATS) diaphragm plication between 2015 and 2022 at our institution were assessed. Functional outcome was analysed with pre- and postoperative pulmonary function tests in sitting and supine position.

**Results:**

We included 43 diaphragm plications, of which 31 were performed via RATS. Morbidity in the RATS- and VATS-cohort were 13 and 8%, respectively (*p* = 0.64), without any major complication (Clavien-Dindo ≥ III, 0%). Surgical time for RATS diaphragm plication was reduced drastically with a median operating time for the first 16 patients of 136 min (range 84–185) and 84 min (range 56–122) for the most recent 15 patients (*p* < 0.0001). Pulmonary function testing after RATS-plication showed a mean increase in vital capacity (VC) of 9% (SD 8, *p* < 0.0001) and of 7% (SD 9, *p* = 0.0009) in forced expiratory volume in 1 s (FEV1) when sitting and 9% (SD 8, *p* < 0.0001) for VC as well as 10% (SD 8, *p* = 0.0001) for FEV1 when in supine position.

**Conclusion:**

RATS diaphragm plication is a very safe and feasible approach, yielding good results in improving patients’ pulmonary function. Further studies are required to elucidate possible advantages over VATS or open approaches.

**Graphical abstract:**

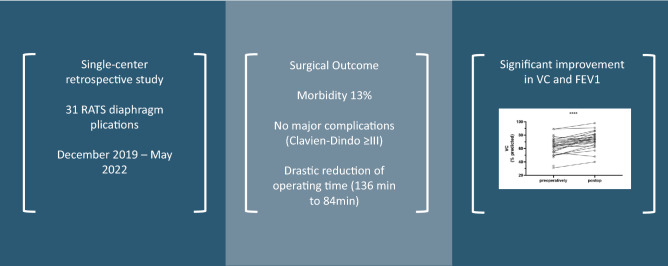

Diaphragmatic paralysis, characterized by the elevation of the hemidiaphragm, can drastically compromise patients’ pulmonary function, leading to dyspnoea, inability to exercise and thus impairment of patients’ quality of life. The most common causes in adults are idiopathic, tumor encroachment of, and trauma to the phrenic nerve, mostly resulting from cardiac surgeries [1]. Diaphragm plication is a well-established treatment and remains the only available treatment to improve pulmonary function. Different techniques including open thoracic, thoracoscopic and laparoscopic approaches have been described previously. The procedure immobilises the hemidiaphragm in a lower position to allow better lung expansion, but also to provide the contralateral hemidiaphragm with a fixed point to pull against, leading to an improved function of the healthy side [2, 3]. Although diaphragmatic paralysis can be an incidental finding in asymptomatic patients, diaphragm plication is only indicated in symptomatic patients.

For open transthoracic diaphragm plication, studies have shown a significant and durable improvement of dyspnoea and pulmonary function of up to 10 years of follow-up [1, 4]. Compared to open approaches, thoracoscopic procedures offer the advantage of less postoperative pain as well as shorter hospitalization. Video-assisted thoracoscopic (VATS) diaphragm plication has widely been reported to also yield excellent results [5]. However, intracorporal suturing in the workspace limited by the rigid ribcage and the elevated hemidiaphragm can be challenging and requires significant expertise. Abdominal approaches offer more workspace and better visualization, as well as avoiding the need for single-lung ventilation and reducing postoperative intercostal nerve pain [6].

In the past 20 years, robot-assisted surgery has been successfully implemented for many thoracic procedures such as lobectomy, thymectomy and mediastinal mass resection. Its three-dimensional magnified imaging as well as great degrees of rotational freedom offer the surgeon excellent visualisation and allow precise and accurate tissue manipulation. Its use for thoracoscopic diaphragm plication was first described in 2012 by Kwak et al. [5]. We have established this method successfully for diaphragm plication in our centre and present our experience with RATS diaphragm plication regarding surgical outcome as well as functional parameters including pulmonary function test (PFT) improvement.

## Patients and methods

### Ethical statements

This study was approved by our local ethics committee (Ethik-Kommission der Albert-Ludwigs-Universität Freiburg, August 30th 2021, No. 21-1515) and registered as a clinical trial (DRKS-ID: DRKS00025829). Written patients informed consent was waived by the ethics committee.

### Patients and methods

This retrospective observational study was conducted at the Department of Thoracic Surgery at the Medical Center—University of Freiburg, Germany. RATS diaphragm plication was established as the standard operative approach in our centre in December 2019, before that VATS was the standard approach. After introduction of RATS diaphragm plication, VATS approach for plication was abandoned after a short transition phase because of the superior visualisation and range of movement for the surgeon with the robotic system.

We included all patients who underwent VATS diaphragm plication between January 2015 and May 2020 or RATS diaphragm plication between December 2019 and May 2022 at our institution.

Patients were selected to undergo plication who presented with lifestyle-limiting dyspnoea, aggravated when swimming, bending over, or in supine position. Chest X-ray and computed tomography of the thorax showed an elevated hemidiaphragm. Thoracic ultrasound showed the diaphragms elevation and/or dysfunction. In addition, body plethysmography, diffusion capacity for carbon monoxide (DLCO) and spirometry in sitting and supine position were performed, based on the standards of the European Respiratory Society (ERS). A decline of 10% of vital capacity (VC) from sitting to supine position confirmed the diagnosis of a restriction of the diaphragmatic function. Postoperatively, lung function was tested again. For more accessible analysis in case of multiple measurements, the superior result was included in the analysis.

The primary outcome parameter for the study was lung-function improvement after surgery, in particular, vital capacity (VC), describing the lung volume difference between maximum inspiration and maximum expiration and forced expiratory volume in 1 s (FEV1), indicating the volume of breath, that, after maximum inspiration, can be exhaled in 1 s. Secondary outcome parameters included morbidity, conversion rate, postoperative complication rate and severity, mortality, surgical time, blood loss, duration of indwelling chest tube and time spend in intensive care, severity of postoperative pain as well as recurrence.

### Surgical method

**Fig. 1:**
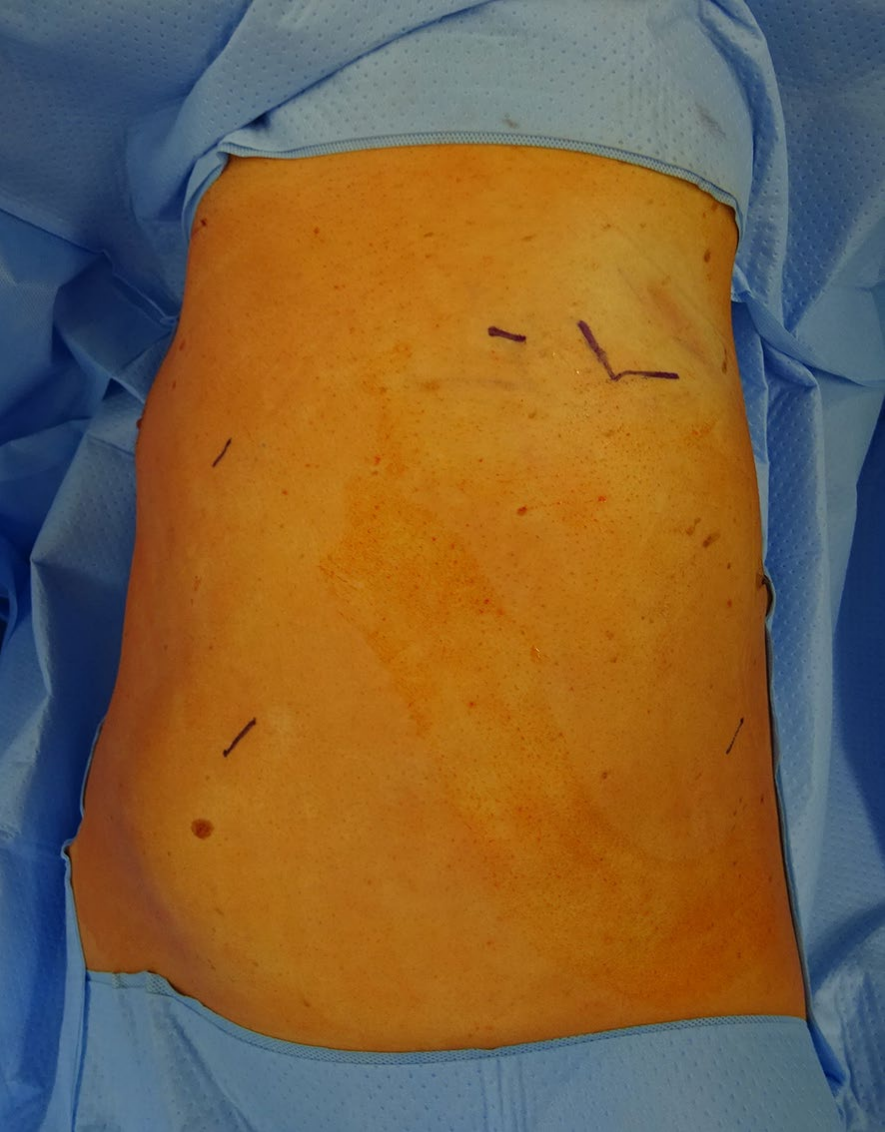
RATS diaphragm plication, patient placement and trocar positions: Patient in lateral decubitus. The 8 mm camera port is placed just anterior to the tip of the scapula. The 12 mm assistant port (fitted with CO_2_-insufflation) is placed between the mid and anterior axillary line just above the diaphragm. Two 8 mm working ports are placed anterior approximately in the 5th intercostal space and posterior as low as possible above the diaphragm

**Fig. 2:**
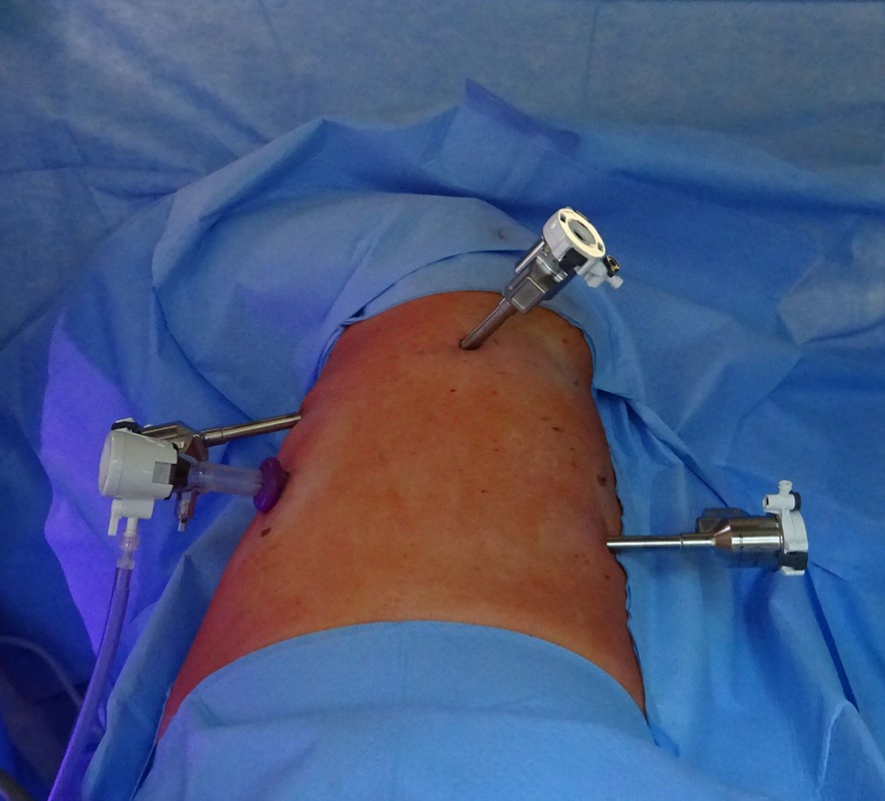
RATS diaphragm plication, patient placement and trocar positions: Patient in lateral decubitus. The 8 mm camera port is placed just anterior to the tip of the scapula. The 12 mm assistant port (fitted with CO_2_-insufflation) is placed between the mid and anterior axillary line just above the diaphragm. Two 8 mm working ports are placed anterior approximately in the 5th intercostal space and posterior as low as possible above the diaphragm

**Fig. 3:**
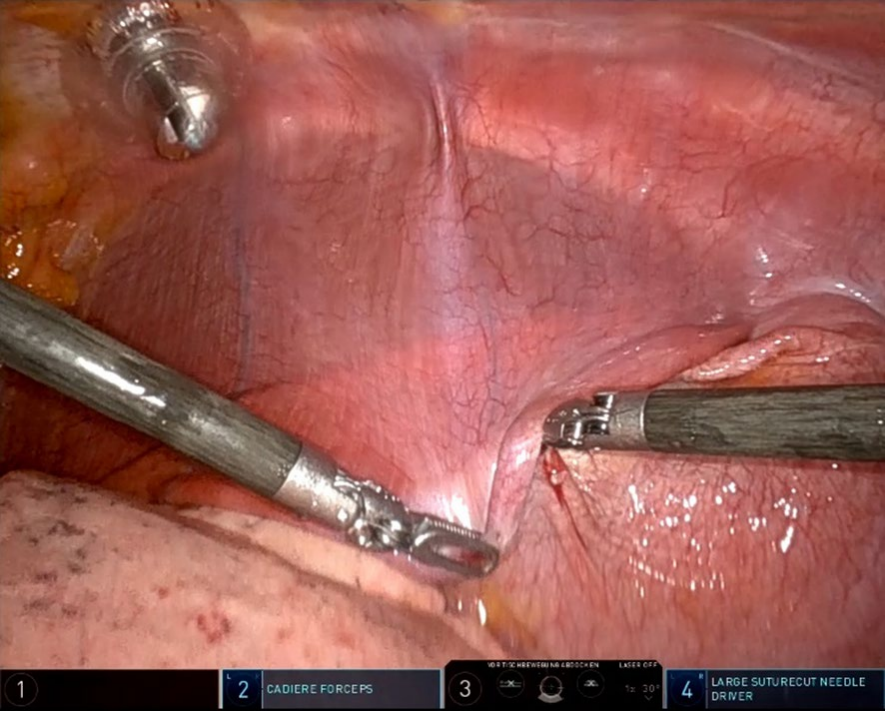
RATS diaphragm plication, thoracoscopic view of the diaphragm: At the beginning of the operation, the diaphragm is grasped and folded to different degrees to visually evaluate the extent of plication

**Fig. 4:**
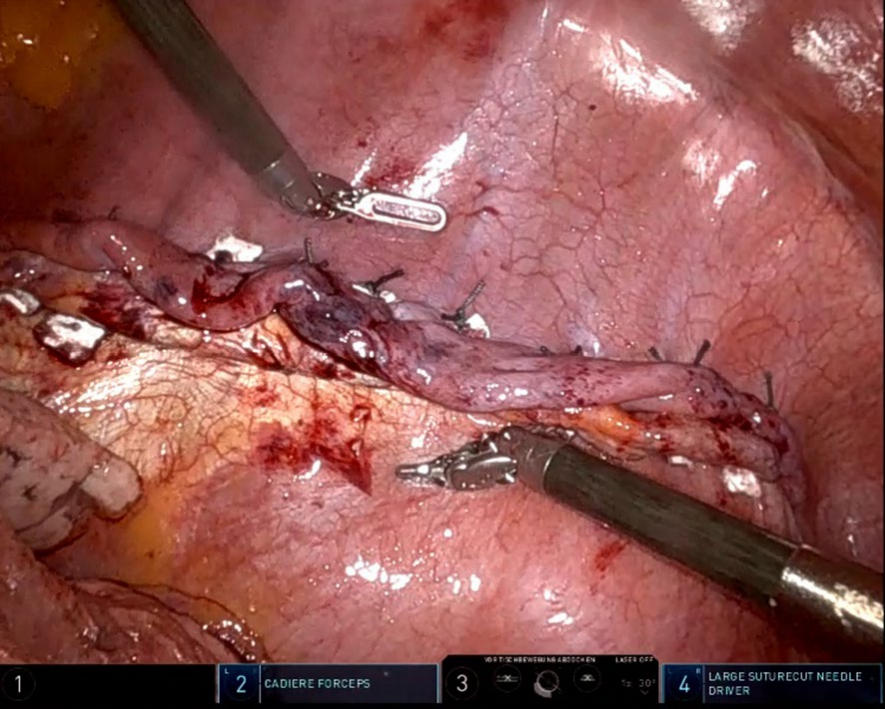
Thoracoscopic view of the diaphragm after RATS diaphragm plication with pledgeted mattress sutures

RATS diaphragm plication was performed with the da Vinci Xi surgical system (Intuitive Surgical, Sunnyvale, CA, USA). Single-lung ventilation was established, and the patient was positioned in the lateral decubitus. Three 8 mm ports and one 12 mm port for the assisting surgeon were placed as seen in Figs. 1 and 2. Depending on the extent of elevation and the intraoperative situs as well as respiratory tolerance, CO_2_-insufflation was used with a pressure ranging from 6 to 10 mmHg as tolerated by the patients to lower the elevated diaphragm and improve visualization. In general, this will push down the diaphragm sufficiently to allow plication. In rare instances the assistant would have to additionally hold down the diaphragm over the assistant port. To evaluate the extent of the plication at the beginning of the operation the diaphragm was grasped and folded to different degrees as seen in Fig. 3 to visually evaluate the extent of plication. Eight to 12 pledgeted 2 ethylene terephthalate sutures (ethibond, non-absorbable, polyfil), depending on the required extend of plication were placed beginning at the center of the diaphragm and continuing medially and laterally as horizontal mattress sutures using the Large Suturecut Needledriver and the Cadiere Forceps. Knots were tied by looping the suture around the instruments similar to tying a surgical knot using instruments during open surgery. A thoracoscopic view of the plicated diaphragm is shown in Fig. 4. By lifting the excess diaphragmatic tissue with the Cadiere Forceps before passing the needle through the diaphragm, sutures could be safely placed without injuring abdominal organs. The size of the needle used was V-37, and pledgets were 7.9 × 7.9 × 1.65 mm. An intercostal block and a 24 Fr chest tube were placed in all patients. After surgery, patients were transferred to our intermediate care ward for postoperative surveillance. Chest tubes were removed depending on output volume and quality after chest-X-ray.

VATS diaphragm plication was generally performed in lateral decubitus with the camera positioned in the 6th intercostal space, lateral of the tip of the scapula and a 4 cm accessory port in the 9th intercostal space in the posterior axillary line. For plication, the excess diaphragmatic tissue was grasped with a clamp and seven to 12 pledgeted 2 ethylene terephthalate sutures were placed in analogy to the technique described above. Postoperative management was similar to that of RATS-plication.

### Statistics

Data were recorded in a database designed in Microsoft Office Excel (Microsoft, Redmond, WA, USA) and GraphPad Prism 9.4.1 (GraphPad Software Inc., La Jolla, CA, USA) was used for statistical analysis. Categorical and count data are presented as frequencies and percentages. In normally distributed data sets paired *t*-test, in non-normally distributed data sets Wilcoxon matched-pairs signed-rank test was performed in case of repeated measurements. For binary variables the Chi^2^-test was applied. In unrelated measurements, Student’s *t*-test and Mann–Whitney-Test were applied, respectively. Results were considered statistically significant if the *p*-value was less than 0.05.

## Results

### Patients

A total of 43 diaphragm plications were included in this study. 31 RATS diaphragm plications were performed between December 2019 and May 2022, of which 17 (55%) were right-sided. 12 patients underwent VATS diaphragm plication from January 2015 to May 2020. In this cohort, 8 plications were right-sided (67%). Male patients accounted for 84% of the patients in the RATS-cohort and 67% in the VATS group. The mean age at surgery was 65 years (SD 11) in the RATS-group, respectively, 69 years (SD 9) in the VATS cohort. Median body mass index (BMI) was 30 in both cohorts (RATS: range 20–41, VATS: range 25–35). The median Charlson-Comorbidity-Index (CCI) was 3 (range 0–6) in the RATS-group, with patients reporting a history of congestive heart failure (*N* = 2, 6%), peripheral vascular disease (*N* = 1, 3%), cerebrovascular accident or transient ischemic attack (*N* = 2, 6%), lung disease (*N* = 6, 19%), diabetes (*N* = 6, 19%) and chronic kidney disease (*N* = 1, 3%). Median CCI in the VATS-cohort was 3 (range 1–4), with patients having a history of lung disease (*N* = 2, 17%) and diabetes (*N* = 2, 17%). Patients’ characteristics and etiology of diaphragmatic paralysis are noted in Table 1.

**Table 1:** Patient characteristics

	RATS	VATS	*p*-value
	(*n* = 31)	(*n* = 12)	
Age, years (mean, SD)	65 (11)	69 (9)	0.16
Female	5 (16%)	4 (33%)	0.24
Right-sided plication	17 (55%)	8 (67%)	0.73
CCI (median, range)	3 (0–6)	3 (1–4)	0.88
BMI (median, range)	30 (20–41)	30 (25–35)	0.73
Etiology			0.53
Idiopathic	19 (61%)	5 (42%)	
Iatrogenic	6 (19%)	3 (25%)	
Trauma	1 (3%)	2 (17%)	
Plexusneuritis	1 (3%)	1 (8%)	
Herniated intervertebral disc	2 (6%)	0	
Neuralgic amyotrophy	1 (3%)	1 (8%)	
Neuroborreliosis	1 (3%)	0	
Operative parameters			
Operating time, min (median, range)	109 (56–185)	101 (47–159)	0.31
Blood loss, ml (mean, SD)	20 (23)	32 (36)	0.51
Conversion to open approach	1 (3%)	0	1
Postoperative course			
IMC, days (median, range)	1 (0–6)	1 (0–3)	0.5
Chest drain, days (median, range)	1 (1–7)	2 (1–4)	0.008
Morbidity	4 (13%)	1 (8%)	0.64
Major complication (CD ≥ III)	0	0	1
Follow-up			
Recurrence	1 (3%)	2 (17%)	0.18

*BMI* Body Mass Index, *CCI* Charlson-Comorbidity-Index, *IMC* Intermediate Care, *CD* Clavien-Dindo

### Surgical outcome parameters

Postoperatively, 4 patients (13%) in the RATS-cohort suffered minor complications (Clavien-Dindo ≤ II; urinary tract infection, postoperative pneumonia, tachyarrhythmia, reflux esophagitis) and none of the patients suffered a major complication (0%, Clavien-Dindo ≥ III). There was one minor complication (8%, postoperative pneumonia) in the VATS group and no mortality in any of the cohorts (morbidity: *p* = 0.64, major complications: *p* = 1).

**Fig. 5:**
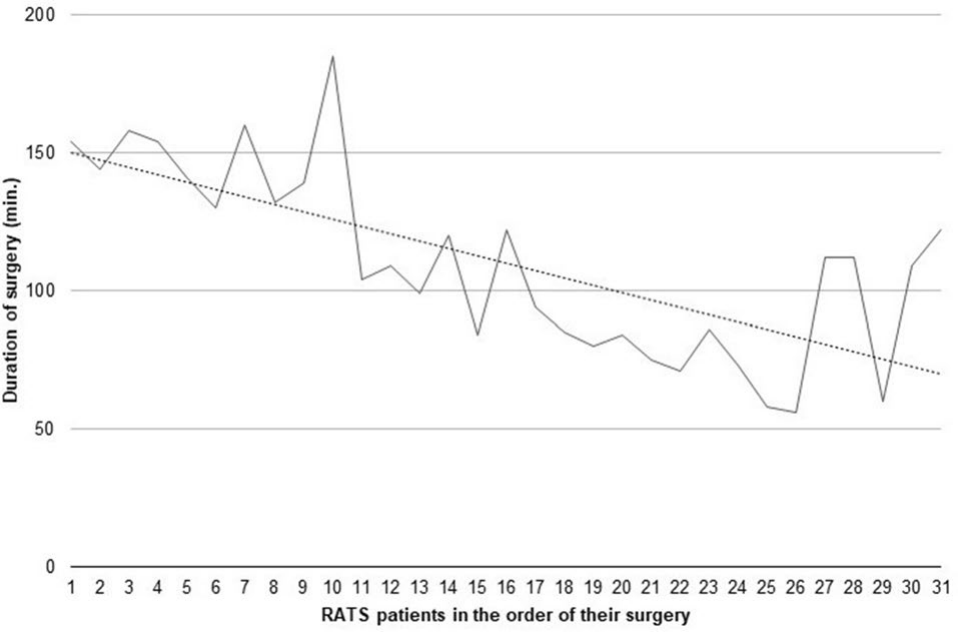
Operating time: The learning curve for RATS diaphragm plication was steep. The median operating time was 109 min (range 56–185) for RATS-plication, with a median operating time for the first 16 RATS patients of 136 min (range 84–185) and 84 min (56–122, *p* < 0.0001) for the most recent 15 RATS-patients. Operating times for patients 27, 28, 30 and 31 do not follow the trend, because a new surgeon was trained for robotic diaphragm plication

The median operating time was 109 min (range 56–185) for RATS and 101 min (range 47–159, *p* = 0.31) for VATS plication, with a median operating time for the first 16 RATS patients of 136 min (range 84–185) and 84 min (56–122, *p* < 0.0001) for the most recent 15 RATS-patients. Operating times are visualized in Fig. 5. It should be noted, that operating times for patients 27, 28, 30 and 31 do not follow the trend line because with these patients, a new surgeon was trained on the robotic system. One patient required conversion from RATS to open surgery (*N* = 1, 3%). There was no conversion in the VATS-cohort (0%, *p* = 1).

The chest tube was removed depending on output volume and quality after a median of 1 day (range 1–7) in the RATS cohort and after 2 days (range 1–4, *p* = 0.008) in the VATS patients. After surgery, patients were transferred to our intermediate care ward for postoperative surveillance. The median stay on IMC was 1 day (range 0–6) in the RATS cohort and 1 day (range 0–3, *p* = 0.5022) in the VATS group. None of the patients required transfer to the intensive care ward.

Post-operative pain was measured with the visual analogue scale (VAS). Patients in the RATS cohort estimated their pain level at 3 in rest (median, range 0–6) and 4 in movement (median, range 1–7) on day 1 after surgery (D1) and at 0 in rest and movement on the day of discharge (median, range in rest 0–3, in movement 0–5), while VATS patients stated a median pain level of 4 in rest and movement on D1 (range each 0–9) and 0 on the day of discharge (range each 0–2). Apart from the length of indwelling chest tube, there was no statistically significant difference in surgical outcome parameters.

### Functional outcome

**Fig. 6:**
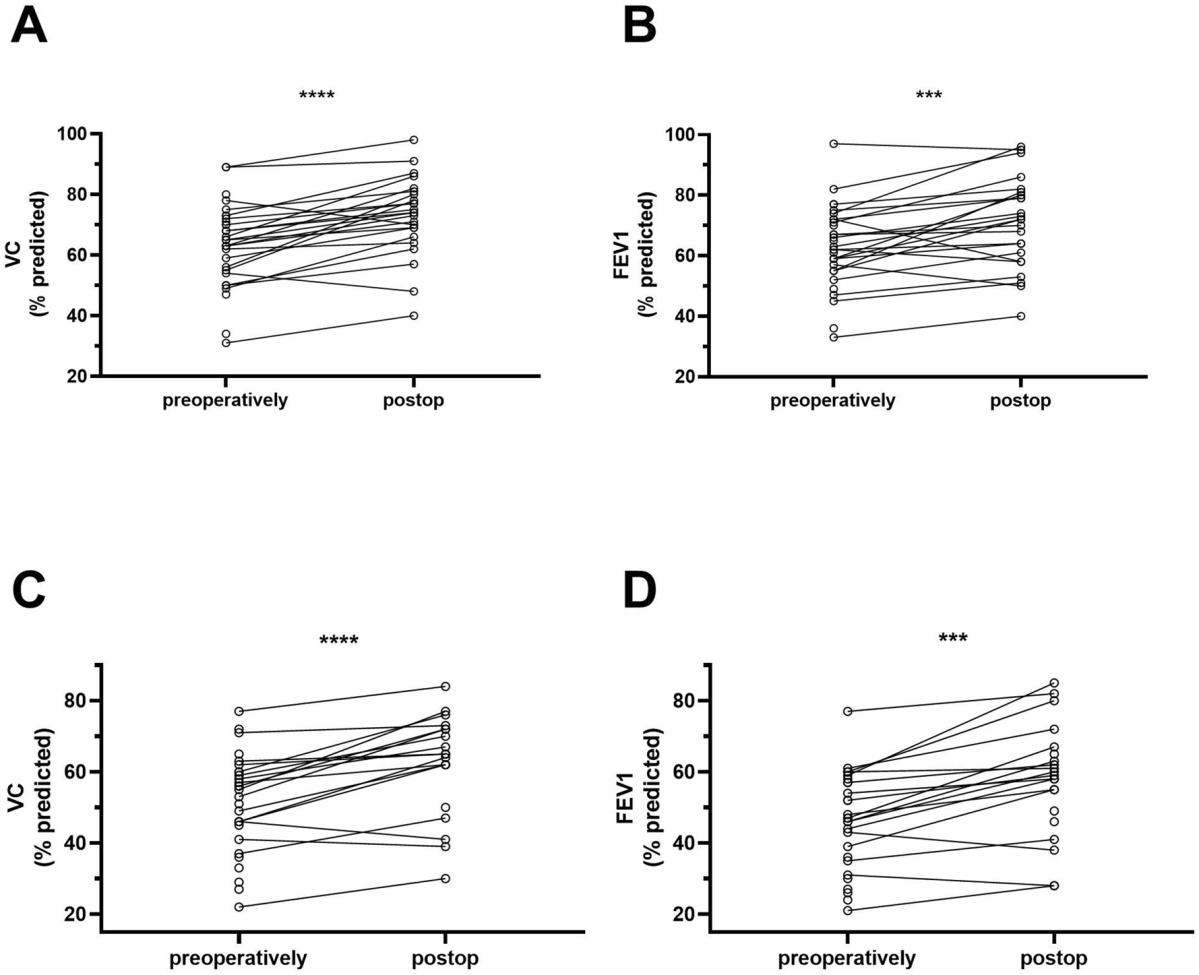
Functional outcome after RATS diaphragm plication: When sitting, mean increase of vital capacity (VC) was 9% (SD 8, *p* < 0.0001) as shown in **A** and mean increase of forced expiratory volume in 1 s (FEV1) was 7% (SD 9, *p* = 0.0009) as shown in **B**. When in supine position, mean increase of VC was 9% (SD 8, *p* < 0.0001) as shown in **C** and mean increase of FEV1 was 10% (SD 8, *p* = 0.0001) as shown in **D**

We could show a mean increase in vital capacity (VC) of 9% (SD 8, *p* < 0.0001) and 7% (SD 9, *p* = 0.0009) in forced expiratory volume in 1 s (FEV1) after RATS diaphragm plication when sitting. Improvement after RATS-plication was 9% (SD 8, *p* < 0.0001) for VC and 10% (SD 8, *p* = 0.0001) for FEV1 when in supine position. This data is visualized in Fig. 6. For VATS, mean improvement in sitting position was 6% (SD 20, *p* = 0.3750) in VC and 8% (SD 15, *p* = 0.3125) in FEV1.

Median follow-up was 12 months (range 1–31 months) for the RATS-cohort and 40 months (range 26–90 months) for the VATS-group. One patient (3%) in the RATS cohort suffered recurrence of diaphragmatic paralysis 18 months after surgery, as did 2 patients from the VATS group (17%, *p* = 0.1836), who had recurrence after 2 and 45 months, respectively.

### Missing values

Due to logistic reasons, not all patients could be followed-up with pulmonary function testing. For the RATS-cohort, preoperative PFT was complete in 28 cases, but PFT in supine position was lacking in 3 patients. 7 patients did not have any postoperative PFT, and another 3 patients were not tested in supine position postoperatively. For the VATS-cohort, one patient had no PFT recorded in our database at all and preoperative PFT in supine position was lacking in 6 patients. One patient did not have any postoperative PFT, and only one patient had postoperative PFT with data from testing in supine position. For more accessible analysis in case of multiple measurements postoperatively, the superior result was included in the analysis and only data of patients with complete pre- and postoperative pulmonary function testing were analysed to measure functional improvement.

## Discussion

Surgical diaphragm plication is a safe and effective procedure regardless of the utilised surgical access [1, 4, 7–11]. In previous studies VATS diaphragm plication was shown to yield similar results in improving PFTs and dyspnoea scores compared to open plication with shorter hospitalisation and lower morbidity and mortality rates [12].

To evaluate possible advantages of the robotic approach we evaluate our first experiences with RATS diaphragm plication, and compare the results to a historic cohort of VATS procedures. Particularly the increased flexibility which facilitates suturing in the confined space and the safer handling of the diaphragm with a robot could improve surgical and functional outcomes.

However, when discussing possible advantages of the robot, financial burden has to be taken into consideration. Besides the procurement cost of the device, used instruments and consumables can be expensive. For robotic diaphragm plication only 2 instruments are needed and thus recurring costs are negligible. Hence, if the device is available, robotic diaphragm plication is a comparatively inexpensive procedure.

Overall, complications were rare in our study and there was no significant difference in morbidity between the RATS and the VATS-cohort. The only statistically significant difference in the two groups were the length of indwelling chest tube, which is most likely due to different surgeons’ preferences. Schumacher et al. reported similar results, with only one patient (7%) requiring treatment for postoperative pneumonia in a cohort of 14 patients undergoing RATS diaphragm plication [11]. In contrast, Asaf et al. reported one patient with paralytic ileus (17%) and one with prolonged air leak (17%) in their 6 patients undergoing RATS, as well as one patient with pleural effusion and acute kidney injury (8%) and one patient with air leak (8%) in their group of 12 patients with transabdominal robot-assisted approach [9].

We could show a steep learning curve for RATS diaphragm plication in our study, with a significant reduction in surgical time; notably, improvement of functional parameters did not differ over time. Others have reported similar observations when establishing robot-assisted approaches, such as Roy et al. who described a median operating time for their first 3 patients undergoing transabdominal robot-assisted diaphragm plication of 255 min and a median operating time of 149 min (range 107–207 min) for the following 19 patients [8]. Considering these results, diaphragm plication seems to be an intervention that is comparatively easy to establish and learn.

Summarizing our observations and the observations made in the most recent studies on diaphragm plication, robot-assisted diaphragm plication was shown to be an extremely safe procedure with minimal morbidity.

Regarding the functional outcome we could show a significant improvement in vital capacity (VC) and forced expiratory volume in 1 s (FEV1) after RATS diaphragm plication. However, improvement was comparatively low in the entire cohort. Previous studies reported a 20% improvement in VC as well as FEV1 6 months after VATS diaphragm plication. This improvement remained constant for a 5-year-follow-up period [7]. In the biggest review on diaphragm plication to date, summarizing data from 13 eligible publications on VATS and open diaphragm plications combining 161 patients, Gazala et al. stated, that improvements in FEV1 and FVC of the order of 20% were to be expected in 97% of patients [12].

### Limitations

Due to logistic reasons and changes in the postoperative follow-up scheme of our institution, postoperative lung-function testing was obtained at varying timepoints and is partially incomplete. In addition, some of the tests were performed only 8 weeks after surgery, which could lead to non-realistic postoperative values after surgery as postoperative pain might impair patients’ ability to participate in pulmonary function testing [14].

There are relatively big differences in PFT improvements in our study, ranging from 10 to 24%, which often did not correlate with the radiologic and most importantly subjective functional results. There are many factors influencing PFT and thus they are a variable parameter and most likely inadequately depict the subjective impairments by diaphragmatic paralysis. Further tools, such as the Cologne Diaphragmatic Paralysis Questionnaire are needed to reliably measure the impact of the disease. This questionnaire specifically addresses subjective health-related impairments in quality of life in patients with unilateral diaphragmatic paralysis and covers physical, functional, psychological, and social aspects [13]. Due to the retrospective nature of our study, application of this questionnaire was not possible for our cohorts.

Additionally, due to small numbers of etiologies other than idiopathic diaphragm paralysis, subgroup analysis was not conclusive in finding significant differences in outcomes of diaphragm plication for different etiologies.

## Conclusion

In summary, RATS diaphragm plication is as an extremely safe procedure with good functional outcome, which is easy to establish and perform. The subjective superiority of the robotic approach was not reflected in the surgical or functional outcome parameters and prospective randomized studies are required to elucidate possible advantages over VATS or open approaches. Other tools than PFT are needed to fully depict the impact of diaphragm plication on quality of life in these patients.

## Abbreviations

BMI: Body mass index
CCI: Charlson-Comorbidity-Index
DLCO: Diffusion capacity for carbon monoxide
ERS: European Respiratory Society
FEV1: Forced expiratory volume in 1 s
PFT: Pulmonary function test
RATS: Robot-assisted thoracoscopic surgery
VATS: Video-assisted thoracoscopic surgery
VC: Vital capacity
